# Outcome after surgical stabilization of symptomatic rib fracture nonunion: a multicenter retrospective case series

**DOI:** 10.1007/s00068-021-01867-x

**Published:** 2022-01-27

**Authors:** Suzanne F. M. Van Wijck, Esther M. M. Van Lieshout, Jonne T. H. Prins, Michael H. J. Verhofstad, Pieter J. Van Huijstee, Jefrey Vermeulen, Mathieu M. E. Wijffels

**Affiliations:** 1 Trauma Research Unit, Department of Surgery, Erasmus MC, University Medical Center Rotterdam, P.O. Box 2040, 3000 CA Rotterdam, The Netherlands; 2 Department of Surgery, Maasstad Ziekenhuis, 3007 AC Rotterdam, The Netherlands; 3 Department of Surgery, HagaZiekenhuis, Els Borst-Eilersplein 275, 2545 AA Den Haag, The Netherlands

**Keywords:** Long-term outcome, Nonunion, Pain, Rib fracture, Surgical stabilization of rib fractures

## Abstract

**Purpose:**

This study aimed to determine the long-term level of pain after surgical treatment of one or more symptomatic rib fracture nonunions. Secondary aims were to evaluate the occurrence of adverse events, satisfaction, and activity resumption. The final aim was to assess the association between pain and the presence of bridging callus at the nonunified fracture. Hypothesized was that thoracic pain would diminish after surgery.

**Methods:**

This retrospective case series included adults who underwent surgery for a symptomatic rib fracture nonunion from three hospitals. Symptomatic nonunion was defined as persistent pain associated with nonbridging callus of ≥1 rib fractures on a chest CT scan at ≥3 months after the initial injury. Patients completed questionnaires about pain, satisfaction, and activity resumption ≥3 months postoperatively.

**Results:**

Thirty-six patients (26 men, 10 women), with a median age of 55 (P_25_–P_75_ 49–62) years and 169 acute rib fractures were included. Nonunion occurred in 98 (58%) fractures of which 70 (71%) were treated surgically. After a median of 11 months (P_25_–P_75_ 7–21), 13 (36%) patients reported severe pain, in contrast to 26 (72%) preoperatively. Patients who underwent intercostal neurectomy or neurolysis in addition to surgical stabilization less often reported pain reduction. Twenty-six (72%) had postoperative complications, for which 12 (33%) underwent additional surgery, mostly for persistent pain. The majority (*n* = 27; 75%) was satisfied with their functional recovery. Of patients who had paid work pre-trauma, 65% had resumed working.

**Conclusion:**

Most patients reported less pain and better daily functioning after surgical stabilization of symptomatic rib fracture nonunions, although causality cannot be proven with this retrospective case series. Additional intercostal nerve treatment was not associated with pain relief. Despite surgery-related complications being common, patient satisfaction was high.

**Level of evidence:** Level V.

**Study type:** Therapeutic.

**Supplementary Information:**

The online version contains supplementary material available at 10.1007/s00068-021-01867-x.

## Introduction

Approximately 10% of blunt trauma patients and almost 50% of polytrauma patients sustain rib fractures [1, 2]. Besides trauma, rib fractures can occur following medical procedures such as cardiopulmonary resuscitation, or ribs can be fractured during coughing especially in patients with low bone density [3, 4]. Whereas most rib fracture patients who undergo nonoperative management recover with no or mild complaints, an unknown proportion develop symptomatic rib fracture nonunion. This is commonly defined as a symptomatic fracture that remains incompletely healed at 3 months or longer post-injury [5, 6]. Rib fracture nonunion can lead to chronic pain, dyspnea on exertion, and rib instability, sometimes accompanied by a clicking sensation. All of these symptoms may negatively impact quality of life [7].

Until recently, it was common practice to surgically resect the symptomatic rib nonunion, creating a defect pseudoarthrosis [8, 9] without aiming for bridging callus formation in the rib fracture. However, bone healing theory suggests that when the mobility of the healing bone exceeds the strain of its tolerance, the fracture will not heal, because no bridging callus can be formed [10]. Consequently, it has been proposed that surgical stabilization of a rib fracture nonunion will provide the correct environment for the bone to heal since it diminishes mobility [11, 12]. Besides insufficient stability, rib fracture healing can be inhibited by soft tissue interposed in the rib fracture. This can be resolved by surgical exploration, potentially promoting bridging callus formation by restoring bone-to-bone contact [12]. Multiple variations have been described for the surgical stabilization of rib fracture nonunions. Differences exist in the technique of surgical stabilization of rib fractures (SSRF) [6, 12–16], the use of bone-grafts [5, 6, 13, 14], and additional treatment of the intercostal nerve [5, 13].

To date, literature addressing the outcomes after surgical treatment of rib fracture nonunion has remained scarce, with the largest study describing 24 patients [5, 6]. These case series reported that pain improved overall and that patients were highly satisfied. Nonetheless, a considerable proportion still experienced persistent symptoms, required additional surgery, and did not return to their pre-injury functional level [5, 6, 13, 14]. This limited amount of data makes it difficult to assess the relative success of current treatments of rib fracture nonunion. Therefore, this study aimed to determine the long-term level of pain after surgical treatment for nonunion of one or more rib fractures. Secondary aims were to evaluate the occurrence of adverse events, satisfaction, and activity resumption. The association of pain with the presence of callus bridging the nonunion of the rib fracture at follow-up and the association of pain with intercostal nerve treatment were also assessed. It was hypothesized that thoracic pain would diminish after surgery.

## Methods

### Setting and study population

This multicenter retrospective case series enrolled adult patients from three hospitals, two level I and one level II trauma center. All three hospitals each have at least 40 admissions of patients who sustained acute rib fractures per year. The surgeons from these three centers (MMEW, PJVH, and JV) have a referral role for complicated rib fracture patients.

All patients aged 18 years or older, with symptomatic nonunion after one or more rib fractures, who underwent surgical treatment between January 1, 2015 and October 31, 2020 were considered eligible. Patients were identified via the hospital records. Symptomatic rib fracture nonunion was defined as pain or discomfort at the site of a rib fracture where the bone failed to unite, without formation of bridging callus, confirmed on a chest Computed Tomography (CT) scan at least 3 months after the initial injury [14, 17]. Patients were excluded if no outcome data were recorded in their medical files, if they sustained the rib fractures spontaneously after radiation therapy or if they had insufficient comprehension of the Dutch language. The Medical Research Ethics Committee of Erasmus MC (MEC-2020–0253) and the local ethics committees of all participating hospitals exempted the study. Informed consent was obtained from all participants.

### Surgical procedure

Preoperative chest CT scans were used to localize the nonunion as an adjunct to physical examination. During the surgical procedure, the nonunion was exposed with attention to the subcostal neurovascular bundle. After debridement of the nonunion, the fracture was fixated. Depending on the hospital, either the MatirixRIB (DePuy Synthes, Amersfoort, The Netherlands), the RibFix Blu™ (Zimmer Biomet, Breda, the Netherlands) or NiTiRib-plate (Nickel-titanium alloy) (Nitinol Rib Clip, Bio Distribution, Luxembourg, Luxembourg) was used for osteosynthesis. Additional techniques were used in case of interposition of tissue, suspected intercostal nerve entrapment, or an abnormal configuration of the ribs. Bone graft was not used. A chest tube was placed in presence of an intra-operative air leak.

### Data collection

Demographics, comorbidities, preoperative analgesic use, and injury details (time between injury, surgery and follow-up, mechanism of injury, number and classification of rib fractures according to the CWIS taxonomy [18], and presence of additional injuries) were collected from the patients’ medical records. In addition, the number of nonunions, surgery details (number and location of treated rib fracture nonunions and additional treatment of the intercostal nerves), occurrence and treatment of adverse events, hospital length of stay, and postoperative imaging results were collected.

Patients were invited at a minimum of 3 months after the nonunion surgery to complete a set of questionnaires on persisting pain, analgesic use, satisfaction with the postoperative results, and activity and work resumption.

### Outcome measures

The primary outcome measure was postoperative thoracic pain at the operated side at final follow-up as determined by a numeric rating scale (NRS) ranging from 0 (no pain) to 10 (worst possible pain). The pain level at follow-up was evaluated for various situations and activities; at night, at rest, during deep inspiration, during high-pressure moments such as coughing or sneezing, and during housekeeping, work, and sports. Preoperative pain at the side with the rib fracture nonunion(s) and the unaffected contralateral side were also scored as a reference.

Secondary outcome measures were the presence of bridging callus in the nonunion, and adverse events including severity, graded according to the Clavien–Dindo classification [19], and their treatment. In addition, patients were asked to complete a questionnaire at the final follow-up regarding analgesic use, work and activity resumption, and satisfaction with the surgical treatment and its results. Satisfaction was determined using an NRS from 0 (very dissatisfied) to 10 (very satisfied). Presence of bridging callus in rib fracture nonunions was assessed with a chest CT scan made at least 3 months after the nonunion surgery. If no postoperative CT scan was performed and the patient reported no complaints during follow-up, presence of bridging callus was assumed in those nonunions.

### Statistical analysis

Data were analyzed using the Statistical Package for the Social Sciences (SPSS) version 25 (SPSS, Chicago, III., USA). Normality of continuous data was tested with the Shapiro–Wilk test. All continuous variables were nonparametric and are presented as median with percentiles. Categorical variables are presented as frequencies and percentages. Missing values were not replaced. To evaluate the association of pain at follow-up with the presence of bridging callus and intercostal nerve treatment in addition to SSRF, group comparisons were conducted using a Chi-squared test. Statistical significance of changes in pain score before and after surgery was assessed using the Wilcoxon signed-rank test.

## Results

During the study period, surgical treatment for rib fracture nonunion was performed in 41 patients, of whom 36 were included; 2 declined to participate, 2 sustained the nonunions after radiation therapy for malignancy and 1 could not be reached (Online Resource 1). The three hospitals enrolled 10, 11, and 15 patients. The median age was 55 years and 26 (72%) were men (Table 1). Three (8%) patients had undergone SSRF for a total of seven acute rib fractures. Three (9%) patients suffered from iatrogenic rib fractures which occurred during a thoracotomy which were performed for a pneumectomy for lung cancer, drainage of pleural empyema, and a Crawford procedure for thoracic-abdominal aortic aneurysm. The other rib fractures were sustained after a trauma (*n* = 26) or coughing/sneezing (*n* = 6). For one patient, the details of the injury, including mechanism and concomitant injuries, were not recorded in the medical files. Of the trauma patients, 15 (58%) sustained also extrathoracic injuries, including 5 with head injuries. Injury Severity Scores were only available in the minority of the patients and therefore not collected. Other baseline characteristics and injury details are shown in Table 1.

**Table 1:** Baseline and injury characteristics for the entire study population

	N*	All patients (*n* = 36)
Age (years)	36	55 (49–62)
Male gender	36	26 (72%)
ASA		
1	36	9 (25%)
2		19 (53%)
3		8 (22%)
Pulmonary comorbidity		
Any	36	7 (19%)
COPD	36	5 (14%)
OSAS	36	2 (6%)
Malignancy	36	1 (3%)
Patients with Previous SSRF	36	3 (8%)
Preoperative analgesic use		
Any	36	20 (56%)
Paracetamol	36	13 (36%)
NSAIDs	36	12 (33%)
Opioids	36	12 (33%)
Anticonvulsants for neuropathic pain	36	1 (3%)
Mechanism of injury		
Fall	35*	11 (31%)
Traffic or sports accident		10 (29%)
Pressure (coughing/sneezing)		6 (17%)
Struck by or against		5 (14%)
Iatrogenic		3 (9%)
Number of acute rib fractures per patient	36	3 (2–5)
Patients with bilateral rib fractures	36	2 (6%)
Patients with multiple (≥3) rib fractures	36	23 (64%)
Patients with flail chest	35*	5 (14%)
Additional thoracic injuries^A^		
Pulmonary contusion	13	3 (23%)
Pneumothorax	19	6 (32%)
Hemothorax	19	9 (47%)
Sternum fracture	26	0 (0%)
Thoracic spine fracture	25	1 (4%)
Scapula fracture	26	1 (4%)
Clavicle fracture	26	0 (0%)
Other additional injuries (AIS ≥ 1)^A^	26	15 (58%)
Body region^A^		
Head	26	5 (19%)
Face		2 (8%)
Neck		0 (0%)
Abdomen		2 (8%)
Spine		1 (4%)
Upper extremity		3 (12%)
Lower extremity		0 (0%)
External		0 (0%)

Data are shown as median (P_25_-P_75_) or as N (%)

*AIS* abbreviated injury severity, *ASA* American Society of Anesthesiologists, *COPD* chronic obstructive pulmonary disease, *NSAID* non-steroidal anti-inflammatory drugs, *OSAS* obstructive sleep apnea syndrome, *SSRF* surgical stabilization of rib fractures

*Provides the number of patients for whom data were available

^a^For traumatic injury mechanisms only (*n* = 26), excluding pressure-induced, iatrogenic, or unknown mechanisms

**Fig. 1:**
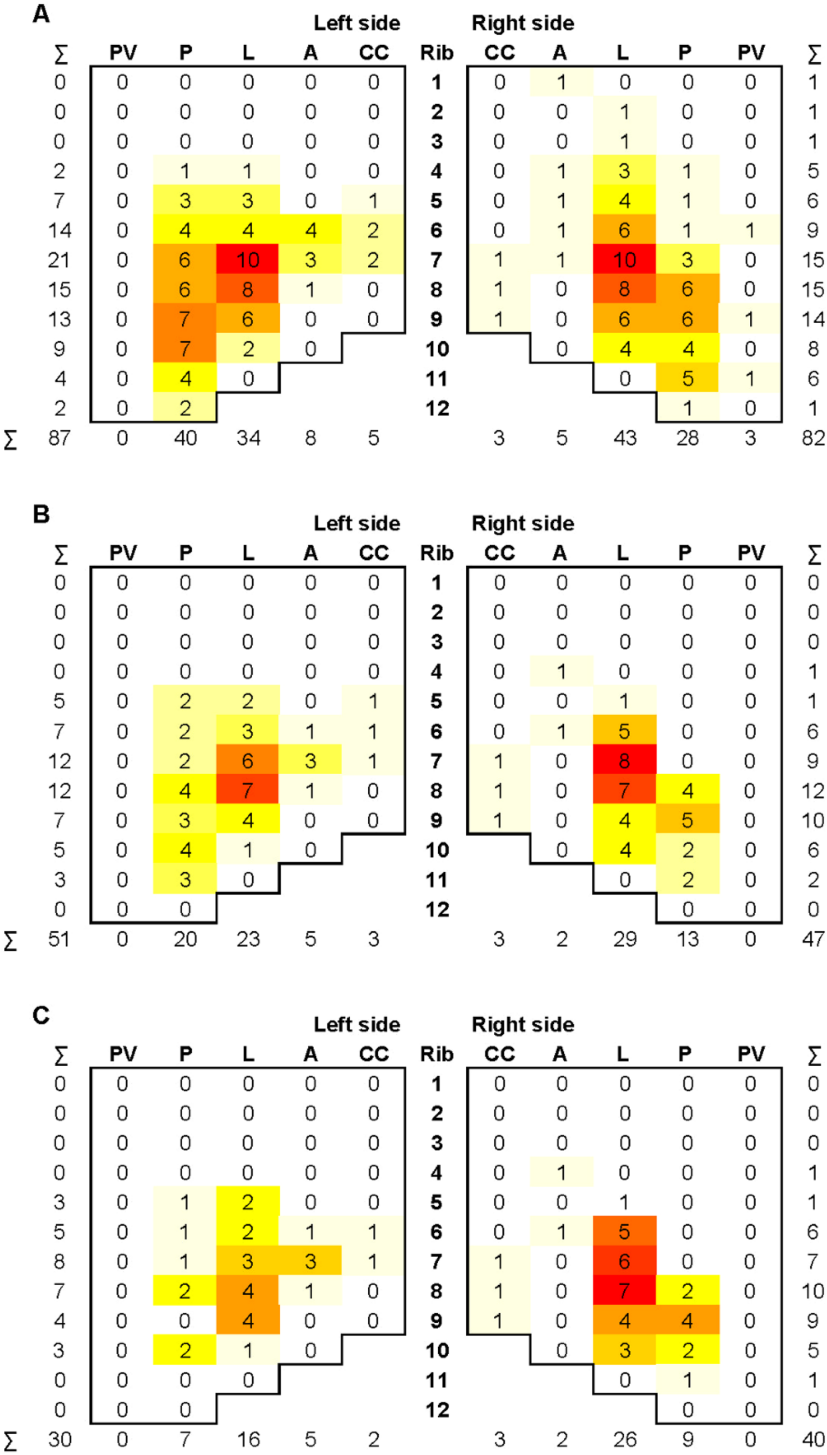
Heatmap showing the number of rib fractures (**A**), rib fracture nonunions (**B**) and surgically stabilized rib fracture nonunions (**C**) per anatomical sector for each rib. *A* anterior, *CC* costochondral, *L* lateral, *P* posterior, *PV* paravertebral

The 36 included patients sustained a total of 169 rib fractures. For 82 of the 169 acute fractures, the initial fracture type and displacement were unknown, because no direct post-injury chest CT was available. For the 87 CT-visualized acute rib fractures, 23 fractures (26%) were diagnosed as displaced with no contact between cortices. The preoperative chest CT scan showed 98 rib fracture nonunions, at a median of 8 months (P_25_–P_75_ 6–12) after trauma. Most of the rib fractures and nonunions occurred laterally and posteriorly in ribs five to ten (Fig. 1a,b). Of the 23 displaced fractures that were visualized on the initial CT, 20 (87%) became nonunions. For the offset and undisplaced fractures, this was a lower percentage; 13 out of 22 (59%) in the offset fractures and 21 out of 42 (50%) in the undisplaced fractures. Twelve (33%) patients had a single rib fracture nonunion. The median number of rib fracture nonunions per patient was two (P_25_–P_75_ 1–4). The maximum number of rib fracture nonunions was six.

The median time between injury and nonunion surgery was 12 months (P_25_-P_75_ 8–19; Table 2). Of the 98 rib fracture nonunions, 70 (71%) underwent SSRF (Fig. 1c). The remaining 28 rib fracture nonunions were not surgically treated, most often because several rib fracture nonunions were not associated with symptoms on physical examination or because they appeared to be sufficiently healed during surgery. In these patients, only intercostal nerve release without SSRF was performed. The maximum number of surgically stabilized rib fracture nonunions per patient was four. Surgical stabilization of rib fracture nonunions was performed using plate and screw osteosynthesis in 22 (61%) patients, and nitinol plates in 12 (34%) (Table 2).

**Table 2:** Nonunion and surgery characteristics for the entire study population

	All patients (*n* = 36)
Ribs with rib fracture nonunion (CT-confirmed) per patient	2 (1–4)
Surgically treated rib fracture nonunions per patient	2 (1–3)
Time between injury and nonunion surgery (months)	12 (8–19)
Surgical stabilization of all rib fracture nonunions	23 (64%)
SSRF to nonunion ratio	1 (1–1)
Osteosynthesis*	
Plate and screw	21 (60%)
Nitinol plate	12 (34%)
Mersiline band + prolene mesh	1 (3%)
Mersiline band + prolene mesh + plate and screw	1 (3%)
Resection of (part of) rib	5 (14%)
Resection of heterotopic ossification	2 (6%)
Intraoperative treatment of intercostal nerve	14 (39%)
Neurectomy	7 (19%)
Intercostal nerve release	6 (17%)
Intercostal nerve infiltration	4 (11%)
Intraoperative AE	
Any	4 (11%)
Problem with fixation	1 (3%)
Pneumothorax	3 (8%)

*In one patient, no osteosynthesis material was placed, because it was impossible to remove the previously placed hardware to access the nonunion. Therefore, the percentages are presented for 35 patients

In 14 patients, the intercostal nerve was treated in addition to stabilization of the rib fracture nonunion by either a neurectomy (*n* = 7), release of the intercostal nerve (*n* = 6), infiltration with a mix of local anesthetics and corticosteroids (*n* = 4), or a combination of these. In two out of three patients with intercostal bridging of heterotopic ossification, the heterotopic ossification was resected. In five patients, a partial rib resection was performed; in two patients to recontour the chest wall and create alignment at the ends of the rib, in three patients to remove infected or necrotic bone. Intraoperative adverse events occurred in four (11%) patients. Three patients developed a pneumothorax and in one patient, it was impossible to remove the hardware applied in an earlier attempt to treat the rib nonunion. The median hospital length of stay was 3 days (P_25_–P_75_, 2–4).

**Fig. 2:**
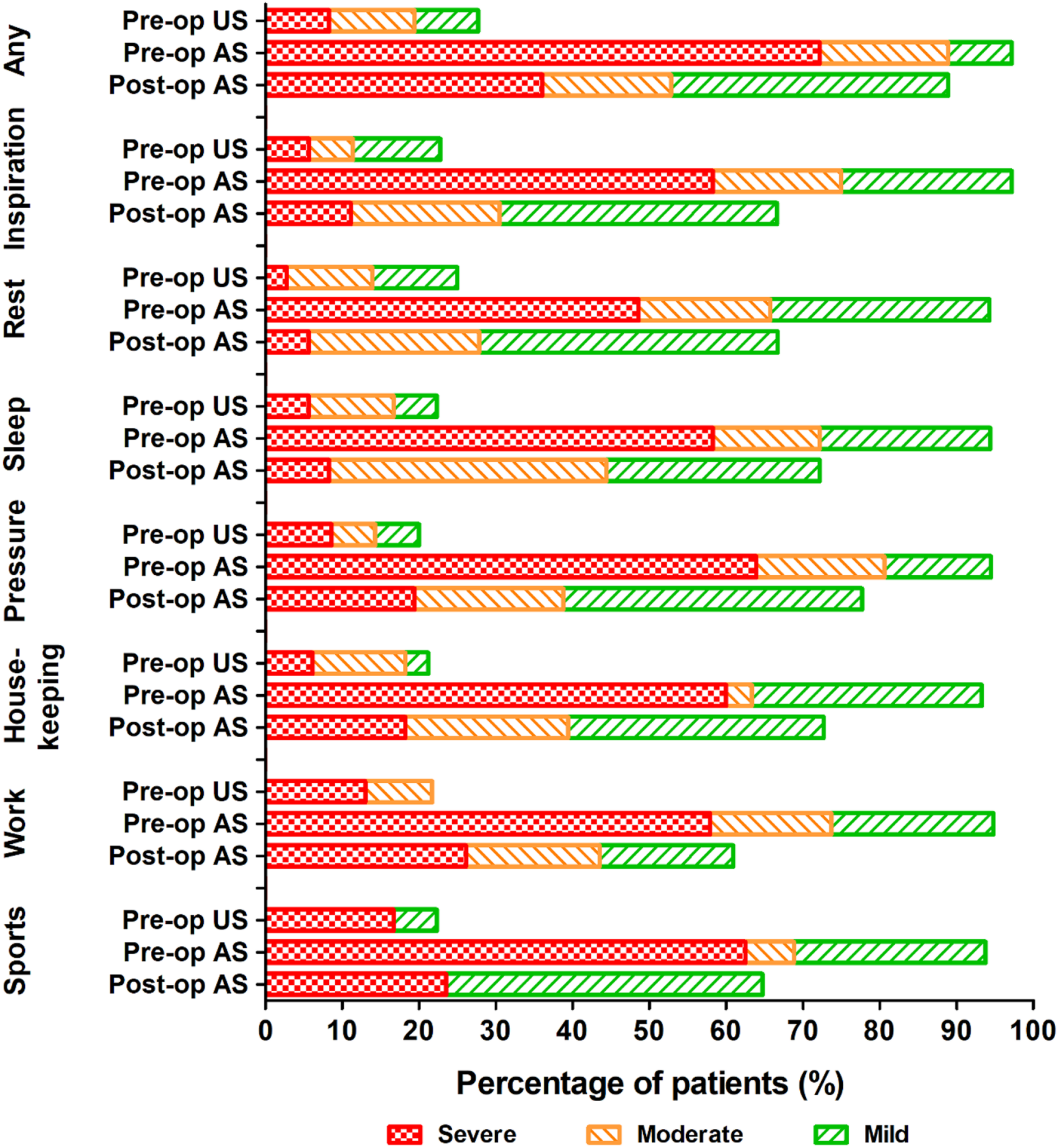
Patient-reported pain level at follow-up during different activities postoperative for the affected side and preoperative for the affected and unaffected side. For number of patients per category, see Online Resource 2. *AS* affected side, *Pre-op* preoperative, *Post-op* postoperative, *US* unaffected side

After a median of 11 months (P_25_–P_75_ 7–21) after the nonunion surgery, 26 (72%) participants reported severe pain before the nonunion surgery, and 13 (36%) reported persistent severe pain at follow-up (Fig. 2 and Online Resource 2). The preoperative and postoperative pain was statistically significantly different for all activities (*p* < 0.041), except for sports (*p* = 0.062). Twenty-one (58%) patients reported less pain at follow-up than before the nonunion surgery. The overall pain remained similar for 12 (33%) and worsened in three (8%) patients. In two patients, this worsening was due to neuralgic pain. In the third in whom the nonunions developed after multiple thoracotomies for pleural empyema, the reason for worse pain was unclear. Two of these three patients were referred to a pain specialist.

Especially painful were the moments of high pressure on the thorax, such as coughing or sneezing, during which 23 (64%) patients reported severe pain on the affected side before the surgery. At follow-up, this diminished but remained painful with seven (19%) patients reporting severe pain after surgery when coughing or sneezing, (*p* < 0.001).

Patients in whom there was bridging callus in all rib fracture nonunions at follow-up more often reported pain reduction (*n* = 19; 73%) than patients without evidence of bridging callus (*n* = 2; 20%; *p* = 0.010; Table 3). Moreover, pain reduction was significantly more often reported by patients who did not undergo neurectomy or intercostal nerve release during surgery (*n* = 19; 76%) than by patients who did undergo intercostal nerve treatment (*n* = 2; 18%; *p* = 0.005). For the other differences in surgical technique, no additional analyses were performed because of the limited number of patients.

**Table 3:** Change in pain at follow-up in patients with versus without evidence of bridging callus in the rib fracture nonunions, or with versus without intercostal nerve treatment

	Bridging callus present*			Neurectomy and/or intercostal nerve release		
	Yes (*n* = 26)	No (*n* = 10)	*P*-value	Yes (*n* = 11)	No (*n* = 25)	*P*-value
Less pain	19 (73%)	2 (20%)	0.010	2 (18%)	19 (76%)	0.005
Similar pain	5 (19%)	7 (70%)		7 (64%)	5 (20%)	
Worse pain	2 (8%)	1 (10%)		2 (18%)	1 (4%)	

Data are shown as *N* (%). There were no missing data

Less pain: highest pain score is  ≥1 category (severe/moderate/mild/no pain) lower than preoperative pain

Similar pain: highest pain score remained in the same category

Worse pain: Highest pain score is  ≥1 category higher than preoperative pain

*Presence of bridging callus is defined as clinical or radiographic (complete or partial) healing of all rib fracture nonunions per patient

In the postoperative course, 26 (72%) patients experienced an adverse event, in which the highest Clavien–Dindo classification was class I in seven patients (19%), II in seven (19%) patients, and IIIb (requiring additional surgery under general anesthesia) in 12 (33%) patients. A detailed list of these adverse events and their clinical management is provided in Table 4. Hardware removal was performed in seven patients (19%), three times for persistent pain on the surgical site resulting in less pain in two patients after removal, and three times because of hardware dislocation with complaints. Other reasons for repetitive surgery were symptoms related to recurrent herniation of lung tissue between the ribs, interposition of muscle between the rib, entrapment of the intercostal nerve in the surgically treated nonunion, and persistent symptomatic nonunion. One patient who sustained the initial rib fracture nonunion while coughing persistently experienced severe pain. A follow-up chest CT scan demonstrated a persistent nonunion of the fixed rib and multiple new fractures without evidence of bridging callus. Therefore, the hardware was removed and additional SSRF was performed of the previously treated rib fracture nonunion and the new nonunions three more times. This patient reported persistent pain at the final follow-up. In two patients, a new fracture occurred at the end of the plate. These fractures showed spontaneous bridging callus formation during follow-up in the outpatient clinic.

**Table 4:** Overview of postoperative adverse events in 26 patients

Clavien–Dindo classification	Specification	*N*
I	Wound problems, including seroma and superficial surgical site infection	7
	Postoperative hematoma	3
	Pneumothorax	2
	New rib fracture in proximity of hardware	2
	Asymptomatic screw dislocation of screw	1
II	Persistent pain requiring treatment by pain specialist	4
	Pathology of intercostal nerve treated nonoperatively	3
	Deep surgical site infection treated with antibiotics	2
	Pneumonia within the first 30 days after surgery	1
IIIb	Persistent pain	
	Requiring hardware removal	3
	Requiring surgical exploration without additional findings	1
	Hematoma requiring surgical evacuation under general anesthesia	2
	Interposition of tissue between ribs requiring additional surgery	2
	Dislocation of screw plate fixation requiring substitution	2
	Dislocation of nitinol plate requiring removal	1
	Persistent symptomatic nonunion	
	Requiring excision	1
	Requiring hardware removal and additional fixation	1
	Entrapment of intercostal nerve, surgically released	1

**Fig. 3:**
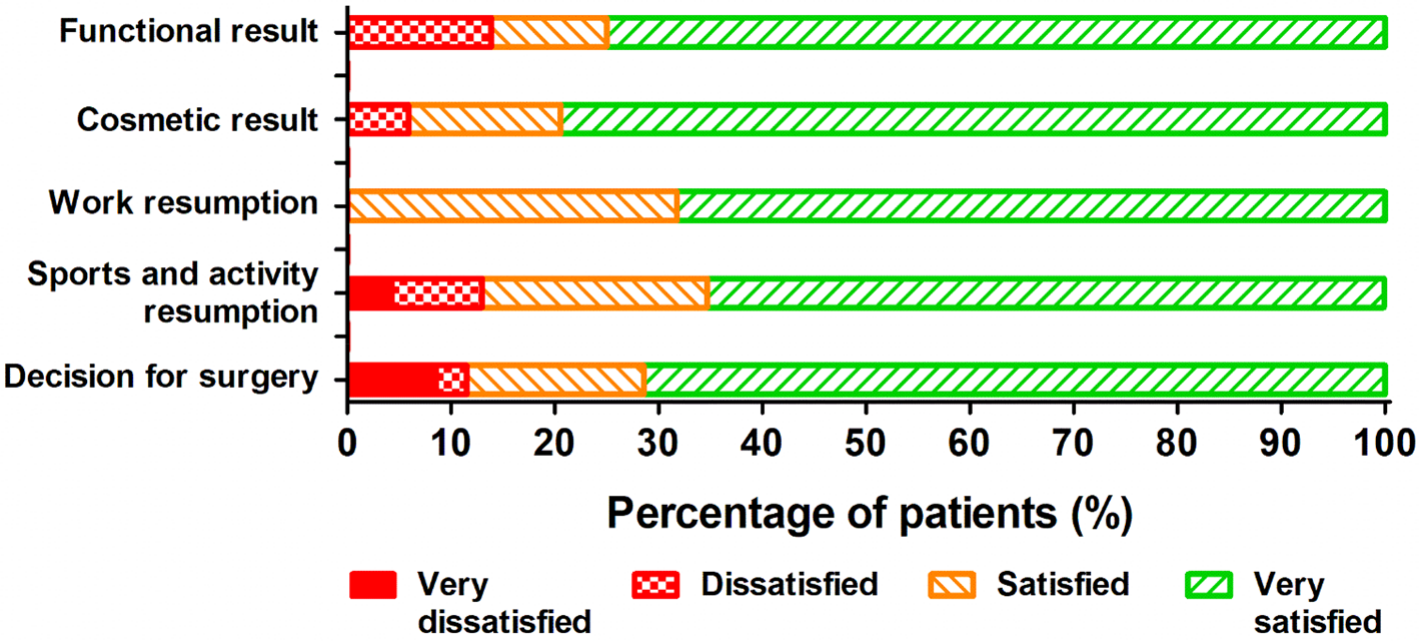
Patient-reported satisfaction at follow-up. For functional result *n* = 36, cosmetic result *n* = 34, work resumption *n* = 22, Sports and activity resumption *n* = 23, decision for surgery *n* = 35

At the final follow-up, analgesics were used by 11 (31%) patients, of whom four (11%) still used opioids. Of the 26 patients who had paid work before surgery, 17 (65%) had fully resumed working, 14 already before the nonunion surgery, and 3 more after the surgery. Before the injury, 16 patients were doing sports. After the nonunion surgery, 13 (81%) resumed their sports activities and 1 started doing sports (Table 5). Overall, 27 (75%) patients were very satisfied with the functional result of the surgery, and 25 (71%) patients were very satisfied with their decision to undergo surgery for the rib fracture nonunion (Fig. 3 and Online Resource 3). Four patients (11%) with persisting symptoms were (very) dissatisfied with the decision to undergo surgery for their rib fracture nonunion.

**Table 5:** Analgesic use and resumption of work and sports at final follow-up for the entire study population

	N*	All patients (*n* = 36)
Analgesic use at follow-up		
Any	36	11 (31%)
Paracetamol	36	9 (25%)
NSAIDs	36	4 (11%)
Opioids	36	4 (11%)
Anticonvulsants for neuropathic pain	36	2 (6%)
Work pre-injury	36	26 (72%)
Work resumption at follow-up	26	
Already resumed preoperatively		14 (54%)
Full		3 (12%)
Partial		2 (8%)
No		7 (27%)
Sports pre-injury	36	16 (44%)
Sports at follow-up	17	
Full or started doing (more) sports		5 (29%)
Partial		5 (29%)
Yes, but other sports		4 (24%)
No		3 (18%)

Data are shown as *N* (%)

The time between surgery and follow-up ranged from 3 to 52 months

*Provides the number of patients for whom data were available

*NSAID* non-steroidal anti-inflammatory drugs

## Discussion

This study evaluated the clinical and long-term outcomes for 36 patients who underwent SSRF for one or more symptomatic rib fracture nonunions. The majority reported improvement of pain compared with before the surgery, especially those in whom there was presence of bridging callus in all the nonunions. It was striking that the results were less good for patients where a neurectomy or release of the intercostal nerve(s) was performed. At follow-up over half of the patients still reported pain and one-third of the patients needed additional surgery, mostly to treat persisting symptoms. Most patients were satisfied with the results and could resume their work and sports.

These findings are consistent with previously published results. In a case series of 18 patients, 56% still experienced moderate or severe pain (NRS ≥ 4) at follow-up, and 32% needed additional surgery [14]. In a second series of 19 patients, 58% experienced pain with movement and 42% remained limited in activities at follow-up; 16% underwent additional surgery [13]. In another case series, two out of ten patients were not able to resume their work or activities due to persisting symptoms and one with a deep infection required additional surgery for debridement and hardware removal [6].

Since most ribs heal after surgical nonunion treatment, sufficient stability seems to be the main goal for the treatment of these nonunions, also because treatment of the intercostal nerve does not seem to improve the outcome. For two (5%) patients in this study, the reason for reoperation was hardware failure. This has been described before in SSRF for nonunion, although there is insufficient literature to evaluate the rate of hardware failure in nonunion SSRF. However, it is larger than the 2% that was previously described in 1224 patients who underwent SSRF for acute rib fractures [20]. A plausible mechanism could be that in these patients, rib nonunions develop not solely due to instability of the fracture, since the plate breaks most likely because of a persistent nonunion. Interposition, low-grade infection, and absence of biologic potential might, among others, play a role in rib fracture nonunion development. If the nonunion appears not to heal after surgery, the treatment strategy should perhaps also focus on these suggested reasons for nonunion development. If the nonunion fails to heal without hardware failure, strain will develop at the end of the plate resulting in iatrogenic fractures in the surrounding area, as was seen twice in this series and as has been previously described [14]. Possibly, different mechanisms of injury have different biomechanical properties in nonunion development. For example, cough-induced fractures might inherently be less stable and could require a different stabilization approach than traumatic rib fractures. Rib fracture nonunions from nontraumatic origin probably warrant a therapeutic strategy that includes optimizing bone quality and extra attention to comorbidities, especially for osteoporosis and malignancies, as was the case for one of the described patients. Unfortunately, methodologically this study could not to evaluate any correlation between mechanism of injury, nonunion development and treatment strategies.

The operative technique varied across the three participating hospitals. Two hospitals used plate and screw osteosynthesis, whereas the third mostly used nitinol plates that lock themselves around the rib when on body temperature. The sample size was insufficient to evaluate differences in biomechanics and outcomes for the different rib fixation systems. Furthermore, various treatments of the intercostal nerves were described. These differences are not surprising, since literature on intercostal nerve treatment during SSRF is scarce [21]. The current sample size was too small to detect differences in clinical and patient-reported outcomes for the various surgical techniques, although it suggests that striving for bridging callus formation in all rib fracture nonunions is associated with a better outcome in terms of pain reduction, which has not previously been reported. In this study, patients who underwent intercostal nerve treatment in addition to stabilization reported more pain than those patients with solitary osseous stabilization, which is not specifically described in previous case series. This suggests that clicking or localized pain, without the need for an intercostal nerve intervention, results in better postoperative results regarding pain. In those patients with more extended, neurological or regional pain the benefit of nonunion surgery might be doubted. Alternatively, intercostal nerve cryoablation could be considered in this group, given the current rise in positive results for the combination of SSRF with cryoablation. In cryoablation, the intercostal nerve bundles are frozen which induces temporary nerve injury known as axonotmesis, resulting in prolonged analgesia [21, 22]. Nevertheless, it should be noted that cryoablation has not been previously investigated specifically in patients with rib fracture nonunion.

Whether the reason for the relatively high patient satisfaction in combination with the large proportion of remaining complaints could be explained by patient selection is unclear. Many patients experienced severe pain for a long period. Patients are counseled preoperatively about the risk of remaining symptoms, and the decision to treat the rib fracture nonunions is reached by shared decision making. This could possibly lead patients to more acceptance of their persistent symptoms after surgery. Also, the fact that even invasive techniques do not solve their problems might result in resignation.

This study has several limitations. First, the retrospective design could have introduced a risk of recall bias, because patients were asked about their preoperative situation after their nonunion surgery, which was at least several months earlier. This risk of recall bias is especially high in patients who sustained their rib fractures during a thoracotomy performed for other reasons. Second, because of the absence of a control group, it is not completely certain that the improvement of pain should be attributed to the surgery, instead of the passing of time alone. Third, there was a wide range in months between surgery and the final follow-up. This variation in passing of time could have led to differences in reported pain at follow-up. In addition, in a substantial number of patients, no chest CT scan was available in the acute setting. This might have led to missed rib fractures or concomitant injuries, resulting in an overestimation of the percentage of rib fractures that became nonunions, which probably is already higher than average due to the patient selection. Furthermore, postoperative imaging was not routinely conducted. Assuming that without postoperative symptoms bridging callus was present might have affected the comparison of pain in patients with and without bridging callus. Bridging callus formation might also have been influenced by variations in the width of the fracture gap that was traversed with the plating system, since the largest fracture gap described was 6 mm after debridement. Moreover, a defect pseudoarthrosis after partial resection of the rib on the nonunion site could also have led to pain relief. Another consideration is that the more rib fracture nonunions per patient, the smaller the chance that all nonunions are healed at follow-up. Finally, biological factors influencing fracture healing including smoking status, vitamin D and parathyroid hormone (PTH) levels were not measured. Despite the limitations, this is the largest cohort study describing outcomes for patients who underwent surgical treatment for one or more symptomatic rib fracture nonunions.

The finding that 87% of the initially displaced fractures were ultimately nonunions, suggests that displacement could be a risk factor for the development of a symptomatic nonunion, whereas the location of the acute rib fractures seemed not to be related to the development of symptomatic nonunions. Future prospective studies with routine imaging could help to identify risk factors for symptomatic nonunion, potentially providing a window of opportunity for prevention by early SSRF [23]. Randomized controlled studies including nonunion patients might list the patient and treatment factors that will lead to the most successful outcome.

## Conclusion

After surgical stabilization of symptomatic rib fracture nonunions, most patients reported pain reduction and improved daily functioning. Causality cannot be proven with this retrospective case series. Although persistent pain and additional surgical interventions are common, surgery is associated with high patient satisfaction. Surgical treatment of the intercostal nerve in addition to SSRF does not lead to relief of symptoms. Future studies on the prevention of nonunion development and optimal treatment strategies of rib fracture nonunions are needed to improve the outcome in these patients.

## Supplementary Information

Below is the link to the electronic supplementary material.

Supplementary file1 (PDF 144 KB)

## Data Availability

Data are available upon request to the corresponding author.
