# Supplementary material for: Outcome after surgical stabilization of symptomatic rib fracture nonunion: a multicenter retrospective case series
Source: Eur J Trauma Emerg Surg. 2022 Jan 27;48(4):2783–93. doi: 10.1007/s00068-021-01867-x (PMC9360056; doi:10.1007/s00068-021-01867-x)
Supplement: Supplementary file 1 — Supplementary file1 (PDF 144 KB) [file 68_2021_1867_MOESM1_ESM.pdf]

**Supplementary information**

**Title:** Outcome after surgical stabilization of symptomatic rib fracture nonunion; a multicenter retrospective case series

**Authors:** Suzanne F.M. Van Wijck[1], MD, Esther M.M. Van Lieshout[1], PhD MSc, Jonne T.H. Prins[1], MD, Michael H.J. Verhofstad[1], MD PhD, Pieter J. Van Huijstee[3], MD, Jefrey Vermeulen[2], MD PhD, Mathieu M.E. Wijffels[1], MD PhD

**Affiliations:**

[1]Trauma Research Unit Department of Surgery, Erasmus MC, University Medical Center Rotterdam, 3000 CA Rotterdam, The Netherlands

[2]Department of Surgery, Maasstad Ziekenhuis, 3007 AC Rotterdam, The Netherlands

[3]Department of Surgery, HagaZiekenhuis, Els Borst-Eilersplein 275, 2545 AA Den Haag, the Netherlands

**Corresponding author:**

Dr. M.M.E. Wijffels

Mail: m.wijffels@erasmusmc.nl

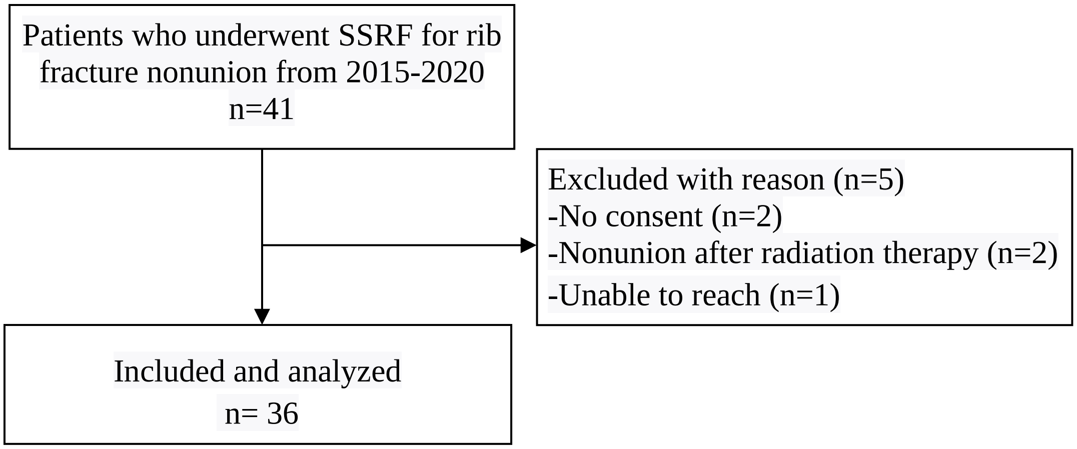


**Online Resource 1. Flowchart of the study**

SSRF, surgical stabilization of rib fracture.

**Online Resource 2. Pain level reported at follow-up during different activities before the rib fracture nonunion surgery for the affected and on the unaffected side, and pain level at follow-up for the affected side**

| | | Before nonunion surgery | | | | After nonunion surgery at follow-up | |
|---|---|---|---|---|---|---|---|
| **Activity** | **Level of pain** | | **Affected side** | | **Unaffected side** | | **Affected side** |
| | | **N** | | **N** | | **N** | |
| Any | No | 36 | 1 (3%) | 36 | 26 (72%) | 36 | 4 (11%) |
| | Mild | | 3 (8%) | | 3 (8%) | | 13 (36%) |
| | Moderate | | 6 (17%) | | 4 (11%) | | 6 (17%) |
| | Severe | | 26 (72%) | | 3 (8%) | | 13 (36%) |
| Inspiration | No | 36 | 1 (3%) | 35 | 27 (77%) | 36 | 12 (33%) |
| | Mild | | 8 (22%) | | 4 (11%) | | 13 (36%) |
| | Moderate | | 6 (17%) | | 2 (6%) | | 7 (19%) |
| | Severe | | 21 (58%) | | 2 (6%) | | 4 (11%) |
| Rest | No | 35 | 2 (6%) | 36 | 27 (75%) | 36 | 12 (33%) |
| | Mild | | 10 (29%) | | 4 (11%) | | 14 (39%) |
| | Moderate | | 6 (17%) | | 4 (11%) | | 8 (22%) |

| | | | | | | | |
|---|---|---|---|---|---|---|---|
| | Severe | | 17 (49%) | | 1 (3%) | | 2 (6%) |
| Sleep | No | 36 | 2 (6%) | 36 | 28 (78%) | 36 | 10 (28%) |
| | Mild | | 8 (22%) | | 2 (6%) | | 10 (28%) |
| | Moderate | | 5 (14%) | | 4 (11%) | | 13 (36%) |
| | Severe | | 21 (58%) | | 2 (6%) | | 3 (8%) |
| Pressure | No | 36 | 2 (6%) | 35 | 28 (80%) | 36 | 8 (22%) |
| | Mild | | 5 (14%) | | 2 (6%) | | 14 (39%) |
| | Moderate | | 6 (17%) | | 2 (6%) | | 7 (19%) |
| | Severe | | 23 (64%) | | 3 (9%) | | 7 (19%) |
| Housekeeping | No | 30 | 2 (7%) | 33 | 26 (79%) | 33 | 9 (27%) |
| | Mild | | 9 (30%) | | 1 (3%) | | 11 (33%) |
| | Moderate | | 1 (3%) | | 4 (12%) | | 7 (21%) |
| | Severe | | 18 (60%) | | 2 (6%) | | 6 (18%) |
| Work | No | 19 | 1 (5%) | 23 | 18 (78%) | 23 | 9 (39%) |
| | Mild | | 4 (21%) | | 0 (0%) | | 4 (17%) |
| | Moderate | | 3 (16%) | | 2 (9%) | | 4 (17%) |
| | Severe | | 11 (58%) | | 3 (13%) | | 6 (26%) |
| Sports | No | 16 | 1 (6%) | 18 | 14 (78%) | 17 | 6 (35%) |

| | | | |
|---|---|---|---|
| Mild | 4 (25%) | 1 (6%) | 7 (41%) |
| Moderate | 1 (6%) | 0 (0%) | 0 (0%) |
| Severe | 10 (63%) | 3 (17%) | 4 (24%) |

Data are shown as N (%). N represents the number of patients for whom data were available.

No pain, NRS 0; Mild pain, NRS 1-3; Moderate pain, NRS 4-6; Severe pain , NRS 7-10

NRS, numeric rating scale.

**Online Resource 3. Satisfaction reported at final follow-up for the entire study population**

| | Functional result (n=36) | Cosmetic result (n=34) | Work resumption (n=22) | Sports and activity resumption (n=23) | Decision for surgery (n=35) |
|---|---|---|---|---|---|
| Very dissatisfied (NRS 0) | 0 (0%) | 0 (0%) | 0 (0%) | 1 (4%) | 3 (9%) |
| Dissatisfied (NRS 1-3) | 5 (14%) | 2 (6%) | 0 (0%) | 2 (9%) | 1 (3%) |
| Satisfied (NRS 4-6) | 4 (11%) | 5 (15%) | 7 (32%) | 5 (22%) | 6 (17%) |
| Very satisfied (NRS 7-10) | 27 (75%) | 27 (79%) | 15 (68%) | 15 (65%) | 25 (71%) |

Data are shown as N (%). N represents the number of patients for whom data were available.

NRS, numeric rating scale.
